# Effect of Allium Extract Supplementation on Egg Quality, Productivity, and Intestinal Microbiota of Laying Hens

**DOI:** 10.3390/ani11010041

**Published:** 2020-12-28

**Authors:** Paloma Abad, Natalia Arroyo-Manzanares, Juan J. Ariza, Alberto Baños, Ana M. García-Campaña

**Affiliations:** 1 DMC Research Center S.L.U., Camino de Jayena n°82, Alhendín, E-18620 Granada, Spain; p.abad@domca.com (P.A.); jariza@dmcrc.com (J.J.A.); abarjona@domca.com (A.B.); 2 Department of Analytical Chemistry, Faculty of Chemistry, Regional Campus of International Excellence “Campus Mare Nostrum”, University of Murcia, E-30100 Murcia, Spain; 3 Department of Analytical Chemistry, Faculty of Sciences, University of Granada, Campus Fuentenueva s/n, E-18071 Granada, Spain

**Keywords:** productivity, intestinal microbiota, laying hen, propyl propane thiosulfonate

## Abstract

**Simple Summary:**

The growing interest in phytogenic products for use in feed, especially in the poultry sector, is mainly due to the improvement in the productivity parameters and gut microbiota modulation properties. For this reason, phytogenic products are becoming excellent candidates as alternatives to the use of antibiotics in animal production to mitigate the negative effects derived from their use. The aim of this study is to explore the ability of allium extract (containing garlic and onion), used as an ingredient in laying hen feed, to improve performance. The promising results obtained in the present study suggest that *Allium* spp. extracts had the potential to be used in feeding laying hens to improve productivity, without affecting egg quality, and to modulate the gut microbiota.

**Abstract:**

The use of allium extract containing propyl propane thiosulfonate (PTSO) as hen feed supplement was evaluated to demonstrate its positive effect on egg production and intestinal microbiota modulation. The study was carried out on 90 laying hens whose feed was supplemented with allium extract for 28 days. Nutritional properties of eggs were not affected, whereas an improvement in productivity was observed based on the increase weight of eggs. In addition, a modulator effect on intestinal microbiota was confirmed by the increase in *Lactobacillus* spp. and *Bifidobacterium* spp., as well as by the reduction in Enterobacteriaceae populations. Finally, the preservation of egg composition was checked by monitoring the content of PTSO, using a new analytical method consisting of the use of solid phase extraction and ultra-high-performance liquid chromatography tandem mass spectrometry (UHPLC-MS/MS). Consequently, based on current results, *Allium* spp. extract rich in organosulfur compounds such as PTSO added to the diet had a beneficial effect on the microbiota and would seem to be a possible alternative to increase productivity, while not affecting the biochemical composition of egg. However, further studies on the effects of allium extract as feed supplement are necessary.

## 1. Introduction

Until their ban in 2006 [1], antibiotic-based growth promoters (AGP) were commonly used as supplements in animal feed due to their benefits in improving digestion efficiency and animal health status [2]. Since then, researchers and nutritionists have shifted their attention toward the search for viable alternative feed additives for supplementary use [3].

Some of the major challenges associated with antibiotic-free poultry production include poor growth performance, lower productivity, and increased morbidity as well as mortality in fowl [4,5,6]. Essential oils (EO) obtained from plants used on livestock and poultry are excellent alternatives to AGP due to their proven positive effects on animal growth and health [7]. A large number of studies have revealed that this type of product could improve the response of the immune system or even enhance productivity [8,9,10,11,12,13].

In recent years, the use of extracts from *Allium* spp. as feed supplement in poultry has been extensively studied [4]. Organosulfur compounds (OSCs) are responsible for the characteristic odor, pungency and biological activities of allium extracts, and these depend on the different *Allium* spp. [14,15,16,17]. Thiosulfinates are the most bioactive OSCs, and are volatile and can easily evaporate, leading to largely varied final concentrations in the feed [18]. Thiosulfinates react quickly in the presence of O2, giving rise to thiosulfonates that are highly stable [14].

The profile of bioactive compounds in allium extract is conditioned by the type of allium variety and specific extraction process [19]. This paper proposes the use of allium extract containing the active ingredient propyl propane thiosulfonate (PTSO) as a feed supplement for laying hens. PTSO is a natural ingredient derived from the natural degradation of the allium flavor precursor named propiin [20]. For broilers, antimicrobial activity of PTSO has been demonstrated in vitro and in vivo against Enterobacteriaceae such as *Escherichia coli* and *Salmonella* spp., as well as against *Campylobacter jejuni* [21,22]. In addition, PTSO could modulate intestinal microbiota composition and improve nutrient digestibility without affecting mucosal enzyme activity in growing broilers [23]. In addition, immunomodulatory effects of PTSO in broilers have been described [12]. The pungent smell of thiosulfonates can affect feed palatability, depending on the applied dosage. In the published literature, the inclusion rates of alliums in poultry have been reported from 10 to 100 mg kg^−1^ [4].

The aim of this study is therefore to evaluate the influence of allium extract intake on laying hens, directly related with PTSO intake, by monitoring egg quality and productivity as well as laying hen health status. For this purpose, a field experiment with 180 laying hens was carried out for 28 days. One half of laying hens (treated group) was supplemented with allium extract, whereas the other half had an allium-free diet (control group). Egg productivity from both groups was compared by studying the number of laid eggs and their weight. In addition, egg quality was determined by the amino acid content and the proximate analysis results. Hen health status was evaluated by feces analyses from each group of microbiota probiotic population (*Lactobacillus* spp. and *Bifidobacterium* spp.), total Enterobacteriaceae count, total anaerobic bacteria, *Clostridium* spp. and *Enterococcus* spp.

Finally, an analytical method was developed to control egg biochemical composition by monitoring PTSO residues [24].

## 2. Materials and Methods

### 2.1. Ethical Statement

This research was carried out at the facilities of farm AVICOLA GARRIDO GARCIA S.L. (Alhendín, Granada, Spain) in real conditions. This research is an observational nutritional trial, in which the slaughtering or direct handling of animals did not occur, reducing their stress during the experiment. This test was carried out following the standard commercial procedure in accordance with Royal Decree 348/2000 [25] and complying with the 1986 European Convention for the protection of vertebrate animals and other scientific purposes. At no time was any animal manipulated. Furthermore, all animal procedures were supervised by the farm′s vets and carried out according to the guidelines of the Helsinki declaration.

### 2.2. Chemical and Reagents

All reagents were of analytical reagent grade. The solvents used as the mobile phase were LC-MS grade. Methanol (MeOH) used as the mobile phase was supplied by VWR BDH Prolabo (Leuven, Belgium). Formic acid (FA) (both analytical and MS grade), ammonium hydroxide 30% (NH_4_OH), potassium sulfate (K_2_SO_4_), sodium chloride (NaCl), potassium chloride (KCl), glutathione reduced 98% (GSH) and cysteine hydrochloride 97% (CYS) were purchased from Sigma Aldrich (St Louis, MO, USA). MeOH, acetone (ACO), acetonitrile (MeCN), ethyl acetate (EtAc) and trichloroacetic acid (TCA) were supplied by Panreac (Barcelona, Spain). Lastly, sodium phosphate dibasic (Na_2_HPO_4_), monopotassium phosphate (KH_2_PO_4_) and peptone solution 1% were purchased from Scharlab (Barcelona, Spain). Ultrapure water (18.2 MΩ cm^−1^, Milli-Q Plus system, Millipore Bedford, MA, USA) was used throughout all of the work.

Clarinert^TM^ 13 mm syringe filters with 0.22 μm nylon membrane (Agela Technologies, Wilmington, DE, USA) were used for sample filtration prior to the injection into the chromatographic system. Extraction cartridges containing a mixed-mode cation-exchange polymeric sorbent (Oasis MCX 150 mg, 6 cc, 30 µm particle size; Waters, Milford, MA, US) were used for SPE. In addition, silica cartridges (Strata SI-1 500 mg, 6 cc, 55 μm particle size, Phenomenex, Torrance, CA) and reversed-phase hydrophilic-lipophilic balance polymer cartridges (Oasis HLB 500 mg, 6 cc; 60 μm particle size Waters) were tested in the optimization of the SPE procedure.

A commercial allium extract (GARLICON P30^®^) rich in PTSO (active ingredient) was supplied by DOMCA Co., Granada, Spain. GARLICON P30^®^ (Feed register number αESP18000180), a powder mix consisting of PTSO (3%) as the active ingredient and sepiolite as the technological additive (97%), was added to hens’ feed at a dose of 1000 mg kg^−1^ (equivalent to 30 mg kg^−1^ of the active ingredient PTSO).

Pure standard PTSO (>90%), was kindly provided by the company DOMCA Co (Granada, Spain). A 2 g L^−1^ stock standard solution of PTSO was prepared by dissolving the appropriate amount of PTSO in MeOH. This standard solution was stored in a dark vial at −20 °C. It was stable for 12 months. Working solutions were prepared diluting the stock solution in MeOH to the desired concentration prior to use. Individual stock solutions of CYS and GSH at concentrations of 10 mM were prepared by dissolving the appropriate amount of each compound in water and stored refrigerated. They were stable for 7 days.

### 2.3. Culture Media Used for Bacterial Growth

The culture media used for bacterial growth analysis were as follows: Slanetz Bartley agar used for the enumeration of *Enterococcus*, MacConkey agar for Enterobacteriaceae total count, Wilkins Chalgren agar for the general growth of anaerobes and sulfite-polymyxin-sulfadiazine agar for enumeration of sulfite-reducing clostridia. All media were provided by Biokar Diagnostics S.L. (Beauvais, France) and subjected to a sterilization process in autoclave at 121 °C for 20 min. Furthermore, a modified Columbia agar medium was used for enumeration of *Bifidobacterium* spp. [26] and a LAMVAB medium was used for *Lactobacillus* spp. count [27].

### 2.4. Instrumentation and Equipment

PTSO analyses were performed using an Agilent 1290 Infinity LC (Agilent Technologies, Waldbronn, Germany) equipped with a binary pump, online degasser, autosampler (5 μL loop) and a column thermostat. Mass-spectrometry measurements were performed on a triple quadrupole mass spectrometer API3200 (AB Sciex, Toronto, ON, Canada) with electrospray ionization (ESI). A Zorbax Eclipse Plus RRHD (50 × 2.1 mm, 1.8 μm, Agilent Technologies) chromatographic column was used for the separation. Instrumental data were collected using the Analysts Software version 1.5 with Schedule Multiple Reaction Monitoring (MRM) ^TM^ Algorithm (AB Sciex).

Amino acid analyses were performed using an Agilent 1260 Infinity LC (Agilent Technologies, Waldbronn, Germany) system consisting of a quaternary pump, online degasser, autosampler (injection range 0.1–900 μL), a column thermostat and photodiode array detector (PAD). Amino acid separation was performed using an AccQ-Tag chromatographic column (150 × 3.9 mm, 4 µm, Waters). A Universal 320R centrifuge (Hettich Zentrifugen, Tuttlingen, Germany), a vortex-2 Genie (Scientific Industries, Bohemia, NY, USA), a polytron (Kinematia AG, Luzern, Switzerland), an oven with natural air convection (Raypa, Barcelona, Spain), a muffle furnace (Selecta, Barcelona, Spain) and an evaporator system (System EVA-EC, from VLM GmbH, Bielefeld, Germany) were also used for sample treatment.

### 2.5. Experimental Animal Design

The field experiment was carried out in a farm located in Alhendín (Granada, Spain). Hens were separated into battery cages with a rate of six animals per cage (experimental unit). Housing temperature was maintained at 20 ± 2 °C with an average relative humidity of 78 ± 3% and a photoperiod (the interval in a 24 h period during which the animal is exposed to light) of 16 h. A total of 180 Lohmann Brown hens (36-week-old) were randomly allocated in two groups defined as control group (G1) and treated group (G2). Each group was constituted by 90 hens and divided in 15 cages.

In a period of 90 days prior to the beginning of the experiment, all hens were given ad libitum access to water and antibiotic-free feed.

The feed was supplied by Piensos Pisur (Peligros, Spain) and homogenized into a helical solid mixer in order to ensure that both G1 and G2 groups consumed a similar feed. Once homogenized, the additive GARLICON P30^®^ was added to the middle of the feed (double mixing procedure). Table 1 summarizes nutritional composition and ingredients information. The additive concentration was determined according to the method described by Abad et at. [28].

The field trial was extended for 28 days (this extension was determined by availability of the farm and the financing of the project). In this period, the access of G1 and G2 to feed was controlled, whereas the access to water was ad libitum. Hens from G1 maintained the standard basal diet, whereas the G2 diet was supplemented with 1000 mg of free n-sulfide compounds allium extract purified (whose main ingredient was PTSO) per kg of feed, which is equivalent to 30 mg of PTSO per kg of feed.

Level values of crude protein, methionine, and lysine are not affected by the addition of garlicon. This was taken into account due the important role they play in laying hen feed [29]. Moreover, methionine and lysine could influence egg weight results [30,31,32].

Several studies in broilers tested dosages of 45 and 90 mg kg^−1^ of PTSO, with its antibacterial activity being demonstrated [22,23]. In addition, anti-inflammatory effects of PTSO have also been demonstrated in vivo at doses among 0.01 and 10 mg kg^−1^ [33]. Based on this information, and in order to maintain level values of crude protein, methionine and lysine, an intermediate dose was selected for finding a compromise between dose and healthy effects.

### 2.6. Egg Productivity

Individual weekly feed intake, egg production and egg weight were recorded. Eggs from both groups were collected weekly, counted and weighed. Egg mass per hen per day was calculated as laying percentage (100 percent = 1 egg per hen per day), multiplied by average daily egg weight. Values per week were calculate by multiplying per 7 (days per week). The feed conversion ratio was calculated from weekly egg mass production and weekly feed intake over 28 days to verify the feed efficiency of each group [34].

### 2.7. Egg Quality

Proximate analysis consisted of determining total fat, moisture, ash, total protein, total carbohydrates and energy content. Total fat in egg samples was determined using the official method of analysis AOAC 925.32–1925 [35]. Moisture was calculated by taking a homogenized portion of egg (5 ± 0.1 g) and heating to 102 °C for 1 h. The percentage of moisture was calculated as [(initial mass-final mass)/initial mass] × 100 [36]. Ash content was determined gravimetrically. An amount of 5 ± 0.1 g of beaten egg was kept in a muffle furnace at 550 °C for 18 h [37]. Ash content was calculated as (ash mass/initial sample mass) × 100. Total protein content was obtained by Kjeldahl procedure using a conversion factor of 6.25 equivalents to 0.16 g of nitrogen per gram of protein [38,39]. Total carbohydrates were obtained gravimetrically as the difference between 100 and the sum of the percentage of total fat, moisture, ash and total protein content [40]. Lastly, energetic value (Kcal) was obtained by multiplying the number of grams of carbohydrates, proteins and fat by 3.68, 4.36, and 9.02, respectively. The sum of all values corresponds to total egg calories [41]. Finally, the egg amino acid profile was determined by HPLC-UV analysis [42].

### 2.8. Microbiological Determinations

Microbiological analysis was performed by fecal sampling. This procedure was selected with the objective of minimizing animal manipulation. Other procedures could be a stressful activity (required to collect feces from the cloaca) and could affect egg laying. Analysis of intestinal content was also discharged since this study was purely observational and animal sacrifice was not considered.

Fecal samples (25 ± 0.1 g per sample) were collected weekly directly from a conveyor belt placed under each cage in sterile containers classified and labeled for analysis. They were diluted with 9 mL of phosphate buffered saline, pH 7.3, prepared as a mixture of different salts at the following concentrations: 8 g L^−1^ NaCl, 0.2 g L^−1^ KCl, 1.15 g L^−1^ Na_2_HPO_4_ and 0.2 g L^−1^ KH_2_PO_4_, and containing CYS in a concentration of 0.5 g L^−1^ to favor anaerobic bacteria survival. Viable counts of bacteria in the feces samples were then estimated by plating serial 10-fold dilutions in the peptone solution. Media and culture conditions to determine each bacterial group are shown in Table 2.

### 2.9. Procedure for the Monitoring of PTSO Residues in Egg Samples

Although several analytical methods for PTSO determination have been established for feed [25,29] or milk [43], PTSO has not been determined as a residue in egg samples. In order to establish a methodology for controlling the sensory attributes of eggs, a new method to analyze PTSO and its derivatives was required. The analytical method for PTSO residues in eggs was developed, taking into account that PTSO reacts in the presence of the thiol group (-SH) of CYS and GSH (both present in egg composition), producing s-propylmercaptocysteine (CSSP) and s-propyl mercaptoglutathione (GSSP), respectively [25]. The aim of that method was to monitor not only PTSO residues but also both of its sub-products to evaluate their possible influence on egg biochemical composition (Tables S1–S3 and Figure S1).

Based on that previous method (the whole information related to the method developed for eggs is included as supplementary information), the analytical procedure applied in eggs is as follows: 5 ± 0.1 g of beaten egg was placed into a 50 mL conical bottom screw tube. Protein precipitation was forced using 10 mL of MeCN with 5% FA and shaken by vortex for 3 min. Samples were centrifuged for 5 min at 5000 rpm and 3 mL of the supernatant solution was percolated through a MCX SPE-cartridge previously conditioned with 3 mL of MeOH. A washing step was then carried out with 4 mL of water. In order to guarantee a total lack of washing solution in the cartridge, a vacuum was applied for a few minutes until no drops were observed falling from the cartridge. The elution step was performed with 4 mL of MeOH solution containing 5% of NH4OH. A volume of 500 μL of the recollected extract was evaporated to dryness under a gentle nitrogen current and the extract was then reconstituted with 1 mL of MeOH:water (1:1, *v*/*v*) solution. Samples were finally analyzed by UHPLC-MS/MS using a mobile phase consisting of 0.05% aqueous FA solution (solvent A), and MeOH (solvent B) at a flow rate of 0.4 mL min−1 (gradient mode). Column temperature was set at 25 °C and the injection volume was 5 μL (full loop). The eluent gradient profile was as follows: 10% B (2.5 min) and 100% B (3 min). It was then reduced back to 10% B in 0.5 min, where it was maintained for 2 min for column equilibration. The MS was working with ESI in positive mode (ESI+) under MRM conditions (Table 3).

**Figure 1:**
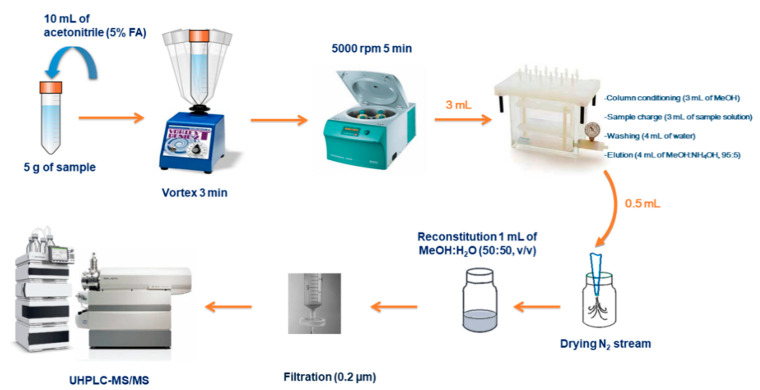
Proposed sample treatment for propyl propane thiosulfonate (PTSO) analysis in eggs.

The target scan time established for each transition was 0.1 s. The ionization source parameters were: source temperature 500 °C; curtain gas (nitrogen) 30 psi; ion spray voltage 5500 V; and GAS 1 and GAS 2 (both of them nitrogen) were set to 50 psi (Abad et al., 2016). Under these conditions, CSSP, GSSP and PTSO were determined in 4.0 min. A scheme of the whole sample procedure is shown in Figure 1.

### 2.10. Statistical Analysis

Statistical analysis was performed by comparing the analyzed variables’ behavior throughout the field trial. The study of the influence of allium extract on egg quality and microbiological analysis was determined by two-factor analysis of variance (ANOVA) establishing time and treatment as fixed effects. In addition, when the interaction was significant, results between G1 and G2 at each time were compared by applying Student’s test (using treatment as fixed effect at 0, 7, 14, 21 and 28 days). All statistical data treatment was performed using STATGRAPHICS Centurion XVI Version 16.2.04 (32 bits).

The experimental unit was the cage. In all statistical analyses, the confidence interval was established at 95% (differences were taken to be significant at *p* ≤ 0.05).

## 3. Results and Discussion

### 3.1. Influence of Allium Extract on Egg Productivity

Feed intake (g per week), egg mass (g per week) and feed conversion ratio during the experimental period are presented in Table 4.

No relevant differences were observed in feed intake per week and hen for G1 and G2 (*p* > 0.05) throughout different time points of the trial (7, 14, 21 and 28 days).

Laid eggs from G1 and G2 were collected, counted, and weighed on a weekly basis. A total of 845 eggs (411 from G1 and 436 from G2) were evaluated throughout the field trial. A sightly increase in egg mass was observed for G2 from the 14th day (*p* = 0.001, 0.011 and 0.034 for the 14th, 21st and 28th day). Therefore, an increase in the average weight was obtained as result of use PTSO as feed supplement.

**Figure 2:**
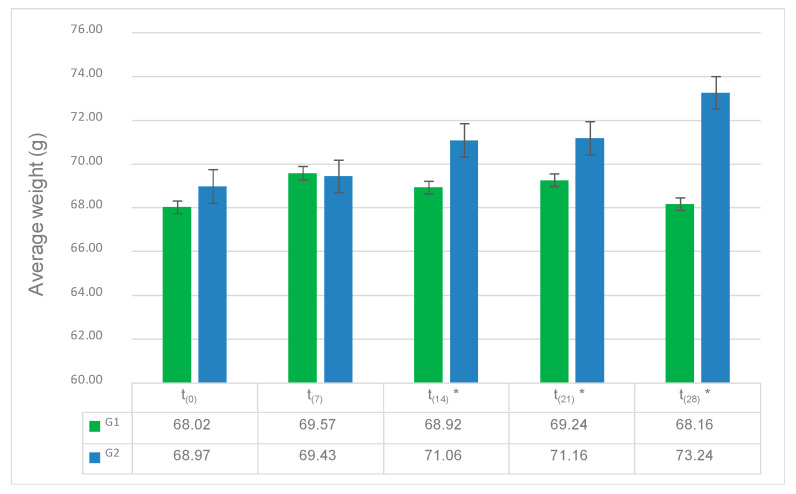
Effect of allium extract (containing PTSO) used as a feed supplement on egg average weight. Results of Student’s test, with G1 being the control group and G2 being the treated group; t_0_ = 0 days; t_7_ = 7 days; t_14_ = 14 days; t_21_ = 21 days; t_28_ = 28 days. Significant effects are represented by *.

The results are shown in Figure 2. As can be seen, a significant increase in egg average weight was observed for G2 from the 14th day and it was extended until the end of the trial (*p* = 0.01, 0.021 and 0.014 for the 14th, 21st and 28th day), as a result of the use of PTSO as a feed supplement. Finally, Student’s *t*-test showed no differences related to the number of laid eggs between G1 and G2 throughout the field trial (*p* > 0.05).

In the same way, there was an impact on feed conversion ratio that was observed by an increase in average weight in G2 by the same feed intake of G1. This trend was observed for the 14th (*p* = 0.005), 21st (*p* = 0.016) and 28th day (*p* = 0.001).

These results may be a consequence of the healthy properties of PTSO (detailed above) when used as a feed supplement, and therefore can be traduced into productive capacity improvement. By having a better profile of the intestinal microbiota and possibly of the general immunity, it is possible to obtain an indirect effect derived from the greater absorption of nutrients and intestinal well-being.

### 3.2. Influence of Allium Extract on Egg Quality

**Figure 3:**
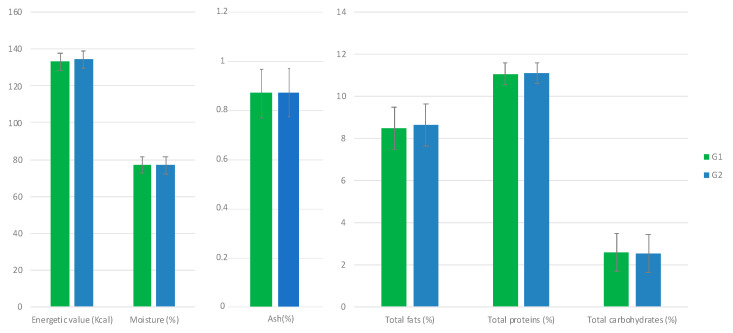
Effect of allium extract (containing PTSO) used as a feed supplement on egg nutritional profile. Results of two-way ANOVA, with G1 being the control group and G2 being the treated group. No significant effects were observed.

The influence of allium extract on egg nutritional parameters was studied by analyzing energetic value, total fat, moisture, ash, total protein and total carbohydrates. No significant differences were seen among time-points of each group, G1 and G2 throughout the trial were observed for egg nutritional parameters, obtaining *p* > 0.05 in all cases. The results obtained for G1 versus G1 by considering all time results (since there is not time dependence) are shown in Figure 3.

**Figure 4:**
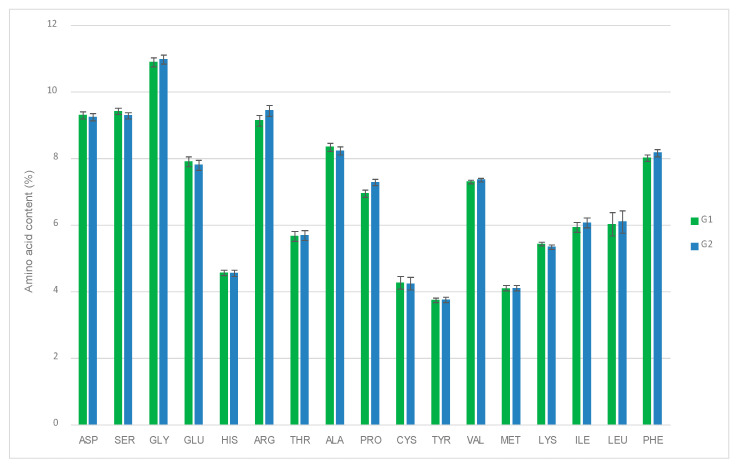
Effect of allium extract (containing PTSO) used as a feed supplement on egg amino acid composition. Results of two-way ANOVA, with G1 being the control group and G2 being the treated group. No significant effects were observed.

Egg amino acid content was also monitored in both experimental groups (G1 and G2) as a quality control parameter. Eggs are characterized by the presence of 17 amino acids: aspartic acid (ASP), serine (SER), glycine (GLY), glutamic acid (GLU), histidine (HIS), arginine (ARG), threonine (THR), alanine (ALA), proline (PRO), CYS, tyrosine (TYR), valine (VAL), methionine (MET), lysine (LYS), isoleucine (ILE), leucine (LEU), phenylalanine (PHE) [44] (Ministry of Agriculture, Food and Environment 2017). Analyses were performed according to the methodology described in the section “Egg quality”. No significant differences among time-points of each group nor G1 and G2 throughout the trial were observed after multivariable analysis (*p* > 0.05 in all cases). The results obtained for G1 versus G1 by considering all time results (since there is not time dependence) are shown in Figure 4 (*p* > 0.05 in all cases).

### 3.3. Influence of Allium Extract on Bacterial Populations in Gut

**Figure 5:**
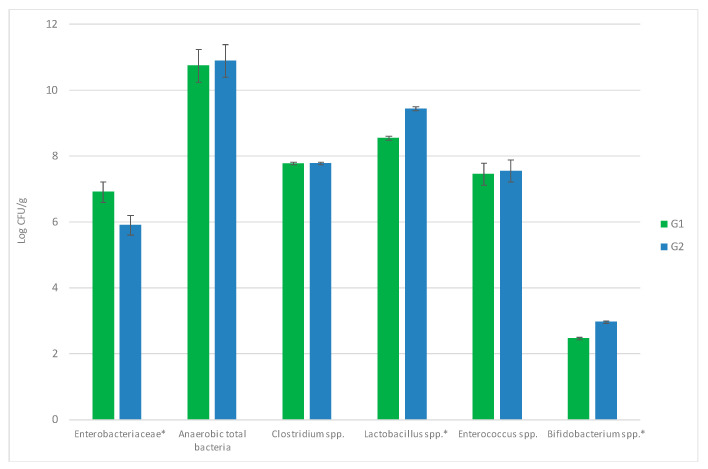
Effect of allium extract (containing PTSO) used as feed supplement on intestinal microbiota microorganisms: Total Enterobacteriaceae, anaerobic total bacteria, *Clostridium* spp., *Enterococcus* spp., *Lactobacillus* spp. and *Bifidobaterium* spp. Results of two-way ANOVA, with G1 being the control group and G2 being the treated group. Significant effects are represented by *.

The effect on intestinal microbiota was studied, since it plays a key role in animal health status and may be influenced by several factors, such as diet, environment, stress or the presence of pathogens [45,46]. The intestinal microbiota (Total Enterobacteriaceae, anaerobic total bacteria, *Clostridium* spp., *Enterococcus* spp., *Lactobacillus* spp. and *Bifidobaterium* spp.) from G1 and G2 was analyzed weekly. No significant differences among time-points of each group nor G1 and G2 throughout the trial were observed for anaerobic total bacteria, *Clostridium spp. and Enterococcus* spp. (*p* > 0.05). The results obtained for G1 versus G1 by considering all time results (since there is not time dependence) are shown in Figure 5.

**Figure 6:**
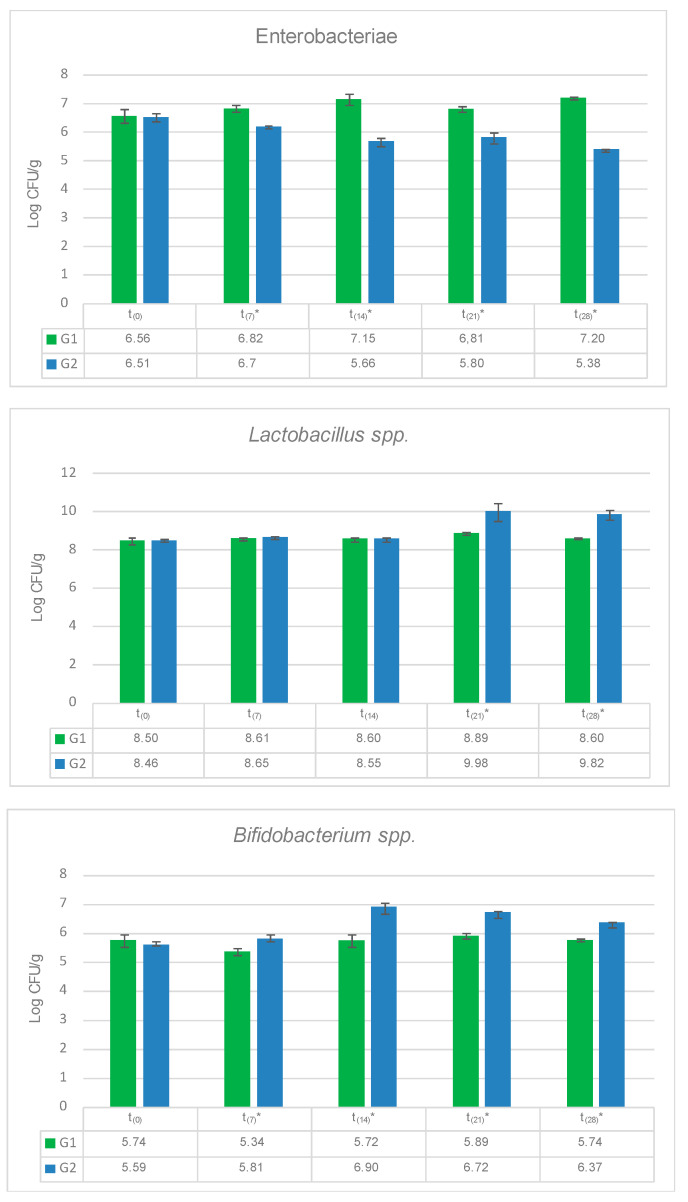
Effect of allium extract (containing PTSO) used as a feed supplement on total Enterobacteriaceae, *Lactobacillus* spp. and *Bifidobaterium* spp. from fecal hens’ samples. Results of Student’s test, with G1 being the control group and G2 being the treated group; t_0_ = 0 days; t_7_ = 7 days; t_14_ = 14 days; t_21_ = 21 days; t_28_ = 28 days. Significant effects are represented by *.

The results obtained for total Enterobacteriaceae, *Lactobacillus* spp. and *Bifidobaterium* spp. confirmed that the use of allium extract containing PTSO as a feed supplement has a significant effect on the intestinal microbiota by reducing the number of Enterobacteriaceae in hen’s feces after 7 days (*p* = 0.028) of treatment (Figure 6). This trend continues until the end of the experiment (days 14th, *p* = 0.020; 21st *p* = 0.029 and 28th, *p* = 0.001). Enterobacteriaceae includes *Salmonella* spp., whose prevention is one of the major challenges in poultry production. PTSO supplementation reduces this pathogen, which could translate to a decrease in associated animal pathologies such as salmonellosis. The reduction in the fecal contamination of eggs ensures animal and food safety [47].

Finally, there was an increase in *Lactobacillus* spp. in G2 compared to G1 at 21st (*p* = 0.011) and 28th days (*p* = 0.035). A similar behavior was observed for total Bifidobacteria population, which increased at the 7th (*p* = 0.041), 14th (*p* = 0.034), 21st (*p* = 0.039) and 28th (*p* = 0.045) days. *Lactobacillus* spp. and *Bifidobacterium* spp. population are associated with the inhibition of pathogen growth. They are also closely related to the increase in nutrient absorption efficiency as well as the improvement of the immunostimulatory effect [48,49]. As can be seen, *Lactobacillus* spp. and *Bifidobacterium* spp. populations were increased by diet supplementation with allium extract. Therefore, it could be said that the presence of PTSO in the diet of laying hens triggers a positive intestinal microbiota modulation. The capacity of allium organosulfur compounds, such as PTSO, to modulate the intestinal microbiota is widely known [21,23]. Recent research reported similar effects in growing-finishing pigs fed with allium extract rich in PTSO, with a significant increase in *Lactobacillus* spp. and a reduction in *Salmonella* spp. and *Clostridium* spp. in feces [50]. Resident gut microbiota members of laying hens also limit and control the colonization of foodborne pathogens. The functional supplementation-feed could strengthen the gut microbiota for improving the performance and colonization resistance to gut pathogens, such as *Salmonella* [51]. The capacity of beneficial gut bacteria to adapt to the exposure of *Allium* spp. derivatives and the evidence that points to the antimicrobial activity against enteropathogens through the organosulfur compounds it contains suggest that this could be a good way to improve gut health in animals [50].

### 3.4. Influence of Allium Extract in Egg Biochemical Composition: Analysis of PTSO Residues in Egg Samples

Twenty egg samples from G1 and G2, respectively, were analyzed weekly. Neither PTSO nor its derivatives (CSSP and GSSP) were detected in any sample. In other words, allium extract residues that could affect egg biochemical composition were not detectable when using the recommended and tested doses.

## 4. Conclusions

The present study has demonstrated that the intake of allium extract containing PTSO as a feed supplement has a positive effect on the productivity of laying hens, increasing egg size and weight.

In addition, evaluation of intestinal microbiota has shown that allium extract reduced Enterobacteriaceae and increased *Lactobacillus* spp. and *Bifidobacterium* spp. populations. In addition, by monitoring possible residues of PTSO or its derivatives in egg samples by UHPLC-MS/MS, this study has proven that the use of allium extract does not alter egg biochemical composition.

## Figures and Tables

**Table 1:** Ingredients of complete feed for laying hens.

Ingredients (in Descending Order): Corn, Roasted Soybean Flour, Wheat, Barley, Fatty Acids, Sunflower Seed Flour, Dicalcium Phosphate, Sodium Chloride, Sodium Bicarbonate.		
Additives	*G1*	*G2*
PTSO (mg kg^−1^)	--	30
Trace element/trace element compounds (mg kg^−1^)		
Manganese (manganese oxide)	65.0	64.4
Zinc (zinc oxide)	37.0	36.6
Copper (cupric sulfate pentahydrate)	4.0	3.9
Iron (ferrous carbonate)	18.0	17.8
Iodine (potassium iodide)	1.9	1.9
Cobalt (basic cobalt carbonate monohydrate)	0.2	0.2
Selenium (sodium selenite)	0.1	0.1
Vitamins (IU kg^−1^)		
Vitamin A, E-672	7.5	7.4
Vitamin D3, E-671	1.50	1.48
Antioxidants (mg kg^−1^)		
Butyldidroxytoluene (BHT), E-321	0.253	0.250
Ethoxyquin, E-324	0.0369	0.0365
Butyldidroxyanisole (BHA), E-320	0.023	0.023
Digestives		
Beta-glucanase endo-1,3 (4) EC 3.2.1.6 (U/kg)	100	99
Beta-xylanase endo-1,4 EC 3.2.1.8 (U/kg)	70	69
4a1640 phytase EC 3.1.3.26 (FTU/kg)	300	297
Colorants (U/kg)		
Canthaxanthin, E-161	3.0	3.0
Beta apocarotenoic acid ethyl ester, E160f	1.6	1.6
Anti-caking agent (mg kg^−1^)		
Sepiolite, E562	600	1560
Amino acids, their salts and analogs (mg kg^−1^)		
Methionine (DL-methionine)	1.837	1.819
Nutrients (%)		
Crude protein	16.0	16.0
Crude fiber	3.9	3.8
Oils and crude oils and fats	3.5	3.5
Crude ash	13.0	12.9
Calcium	3.9	3.9
Phosphorous	0.60	0.59
Sodium	0.20	0.20
Methionine	0.46	0.46
Digestive Lysine	0.97	0.96
Carbohydrates	57.6	57.0

**Table 2:** Culture conditions and growth media used for bacterial growth analysis.

Growth Media	Bacterial Group	T (°C)	Culture Conditions
MacConkey agar	Enterobacteriaceae	35 ± 1 °C	18–24 h aerobic atmosphere
Wilkins-Chalgren	Total anaerobic bacteria	35 ± 1 °C	24–48 h anaerobic atmosphere
Sulfite-Polymyxin-Sulfadiazine agar	*Clostridium* spp.	35 ± 1 °C	24 h anaerobic atmosphere
LAMVAB	*Lactobacillus* spp.	35 ± 1 °C	72–120 h aerobic atmosphere
Slanetz Bartley agar	*Enterococcus* spp.	37 ± 1 °C	24–48 h aerobic atmosphere
Modified columbia agar	Total bifidobacteria	37 ± 1 °C	48–72 h anaerobic atmosphere

**Table 3:** Monitored ions of the target analytes and MS/MS parameters.

Compound	Retention Time (Min)	Precursor Ion (*m/z*)	Molecular Ion	DP ^1^	EP ^1^	CEP ^1^	Product Ions ^2^	CE	CXP ^1^
CSSP	1.7	196.0	[M + H]^+^	36.0	6.0	12.0	107.0 (Q)	11.0	4.0
							179.0 (I)	17.0	
GSSP	1.9	382.0	[M + H]^+^	31.0	9.5	38.0	130.0 (Q)	24.0	4.0
							150.0 (I)	27.0	
PTSO	4.0	183.0	[M + H]^+^	21.0	10.5	10.0	141.1 (Q)	13.0	4.0
							99.0 (I)	19.0	

^1^ DP: declustering potential; EP: entrance potential; CEP: collision cell entrance potential; CXP: collision cell exit potential; CE: collision energy. All expressed in voltage. ^2^ Product ions: (Q) Transition used for quantification, (I) Transition used to confirm the identification.

**Table 4:** Feed intake, hens laying percentage, egg mass and feed conversion ratio values for G1 and G2.

Parameter	G1	G2	*p*
Feed intake (g per day)	154	150	>0.05
Hens laying percentage (%)	91	97.3	>0.05
Egg mass (g per day)	62.79	69.29	<0.05
Feed conversion ratio	2.45	2.16	<0.05

## Data Availability

The data presented in this study are available on request from the corresponding author.

## Supplementary Materials

The following are available online at https://www.mdpi.com/2076-2615/11/1/41/s1, Table S1; Study of the SPE procedure for the extraction of derivatives from PTSO residues (CSSP and GSSP) in egg using different sorbents, Table S2: Statistical and performance characteristics for the SPE-UHPLC-MS/MS proposed method for the determination of PTSO residues, Table S3: Precision (% RSD of peak areas) of the proposed method for the monitoring of PTSO residues in eggs, Figure S1: Chromatogram of an egg sample from a free farming hen spiked with 2.5 mg kg^−1^ of PTSO and subjected to the proposed method.
