# Supplementary material for: Effect of Allium Extract Supplementation on Egg Quality, Productivity, and Intestinal Microbiota of Laying Hens
Source: Animals (Basel). 2020 Dec 28;11(1):41. doi: 10.3390/ani11010041 (PMC7823882; doi:10.3390/ani11010041)
Supplement: Supplementary file 1 [file animals-11-00041-s001.pdf]

animals

MDPI

Article

Effect of Allium Extract Supplementation on Egg Quality, Productivity, and Intestinal Microbiota of Laying Hens

Paloma Abad [1], Natalia Arroyo-Manzanares [2,*], Juan J. Ariza [1], Alberto Baños [1] and Ana M. García-Campaña [3,*]

Supplementay Materials:

Analysis of PTSO Residues in Egg Samples: method development

Optimization of Sample Treatment.

All the experiments were performed using samples of 5 ± 0.1 g of beaten eggs from battery cage farming hens spiked with 200 mg kg^{-1} of PTSO (500 µL of PTSO stock standard solution). In order to achieve full homogenization, the sample was placed in a conical bottom screw tube and it was vortexed for 1 min after PTSO addition. A wide number of methods for protein precipitation in egg have been development in order to remove sample interferences (Trziszka et al. 2013). Acids like TCA or organic solvents are well-known protein-precipitating agents (Sivaraman et al. 1997; Rajalingam et al. 2009). In this work, for PTSO, CSSP and GSSP determination, protein precipitation was carried out by sample centrifugation upon adding a protein precipitation agent. Firstly, several precipitation solvents were tested using a volume of 10 mL in all cases: 20% of TCA in water, MeOH: H_2O (1:1) with 20% of TCA, MeOH, ACO, MeCN and EtAc. In any case, PTSO was found in the final extract, demonstrating that PTSO reacts completely with the amino acids, giving CSSP and GSSP, and the optimization of sample treatment was therefore focused on the extraction of these two corresponding derivatives. Best results in terms of signal intensity were obtained for MeCN. Once MeCN was added to the egg samples, they were shaken before centrifuging. Vortex (1–3 min) and a polytron (1–3 min) were checked as agitation mechanisms and the most efficient in terms of signals was vortex used during 3 min.

Egg is a very complex matrix, hence a SPE procedure was proposed for sample clean-up. Different SPE cartridges were tested in order to evaluate the best option to retain CSSP and GSSP: Silica, MCX and HLB. The applied protocols of conditioning, loading and elution are shown in supplementary data (Table S1).

In order to evaluate the SPE extraction efficiency, a standard solution of CSSP and GSSP at 200 mg L^{-1} was prepared in MeCN for Silica and in MeCN with 5% FA for MCX and HLB procedures. After elution, in all cases, 1 mL was evaporated to dryness under a gentle nitrogen current and reconstituted with 1 mL of injection solvent (MeOH:water, 1:1, v/v). Since no dilution or preconcentration were carried out in any of the evaluated SPE protocols, recoveries were calculated as [(sum of signal of CSSP and GSSP in the final extract / sum of the signal of a standard solution of CSSP and GSSP at 200 mg L^{-1})×100].

The highest recovery percentages (Table S1) were obtained using MCX which was therefore selected as optimum. Subsequently, the optimization of the different parameters affecting the SPE extraction process was carried out in egg sample in order to get the best efficiency in the extraction of CSSP and GSSP. Conditioning solvent volume of MeOH was studied from 3 to 5 mL and no significant influence on the recovery percentage was observed, so 3 mL (minimum volume recommended by the supplier) of MeOH was chosen as optimum. The influence of FA addition in the extraction solvent (MeCN) was also studied between 0–10% and better retention was obtained for 5% of FA, so it was selected as optimum. Lastly, load sample volume was investigated between 3–5 mL. It was observed that as much higher the sample loading volume was, worse results, in terms of recovery percentages were obtained. This could be due to the presence of matrix components which could saturate the MCX column. Therefore, best signal intensity was obtained for 3 mL and this volume was used as optimum.

The introduction of a washing step was evaluated in order to obtain a cleaner extract and consequently reduce the matrix effect. Aliquots of 3 mL of water with different concentrations of FA (0%, 1%, 2% and 3%) were tested and a decrease in the analytical signal was observed when the percentage of FA was increased. Apparently, no significance differences were obtained when introducing the washing step using water in terms of peak area. However, it was applied in order to achieve a reduction of matrix effect and a better signal to noise ratio.

Subsequently, the volume of water used in the washing step was evaluated between 2–5 mL, and 4 mL were selected. Because of the high cartridge volume, it was necessary to apply vacuum after the washing and elution steps in order to remove the washing solvent from the cartridge before elution. Also, the influence of the elution solvent volume was also studied between 1–4 mL, and 4 mL of $MeOH:NH_4OH$ (95:5) were selected as the best option in order to elute efficiently all the retained compounds.

Lastly, the volume of extract to be dried was set at 500 L since higher or lower volumes involved an increase in matrix effect or further dilution, respectively. The extract was evaporated to near dryness using a gentle stream of N_2, the residue was reconstituted with 1 mL of MeOH:water (50:50). A summary of the final sample treatment is included in section Analysis of PTSO residues in egg samples and a chromatogram of an egg sample submitted to the optimized sample treatment is shown in Figure S1.

Characterization of the SPE-UHPLC-MS/MS.

In order to check the suitability of the method for the monitoring of possible residues of PTSO, a full characterization was carried out for eggs of category 3 (battery cage farming), type of eggs obtained from the animal trial.

For the characterization of the proposed method, different parameters such as linear dynamic range, determination coefficient (R^2), limit of detection (LOD) and limit of quantification (LOQ) and precision were evaluated for each matrix. PTSO residues were monitored as sum of its derivatives (CSSP and GSSP).

Matrix-matched calibration (MMC) curves were obtained using egg samples spiked at six different concentrations of PTSO (from 0.25 to 25 mg kg^{-1}). Each concentration level was prepared in duplicate, submitted to the subsequent sample treatment and injected in triplicate. Sum of CSSP and GSSP peak area was considered as analytical signal. Prior to that, blank egg samples from laying hens whose diet was PTSO-free were analyzed in order to check a possible presence of PTSO, CSSP or GSSP and none of them gave a positive result. Statistical parameters were calculated by least-square regression, while LODs and LOQs were calculated as 3× S/N ratio and 10× S/N ratio, respectively. The results are summarized in Table S2. The satisfactory determination coefficients confirm that PTSO analytical responses were linear over the studied range.

The precision of the whole method was evaluated in terms of repeatability (intraday precision) and intermediate precision (interday precision). Repeatability was assessed for samples from the two studied egg types by application of the whole procedure on the same day to three samples of each kind (experimental replicates) spiked at three concentrations levels of PTSO (0.250, 2.5 and 25 mg kg^{-1}). Each extract was injected in triplicate (instrumental replicates). Intermediate precision was evaluated with a similar procedure, by spiking and analysing samples injected in triplicate, during five different days. The results, expressed as relative standard deviation (% RSD) of peak areas are shown in Table S3. As it can be seen, satisfactory precision with RSD lower than 10 % was obtained.

Supplementary references

Rajalingam D, Loftis C, Xu J and Kumar T. Trichloroacetic acid-induced protein precipitation involves the reversible association of a stable partially structured intermediate. *Protein Sci.* **2009, 18**, 980–993..

Sivaraman T, Kumar TKS, Jayaraman G and Yu C. The mechanism of 2,2,2-trichloroacetic acid-induced protein precipitation. *J. Protein Chem.* **1997,** *16*, 291–297.

Trziszka, T., H. Różański, Polanowski, A.. Eggs as a very promising source of biomedical and nutraceutical preparations: a review. *J. Life Sci.* **2013** ,*8*, 862–877.

animals

MDPI

Supplementary Tables

Table S1. Study of the SPE procedure for the extraction of derivatives from PTSO residues (CSSP and GSSP) in egg using different sorbents.

Parameter	Silica	MCX	HLB
Conditioning (3 mL)	Hexane	MeOH	MeOH/H_2O
Loading (4 mL)	Sample solution in MeCN	Sample solution in MeCN (5 % FA)	Sample solution in MeCN (5 % FA)
Elution (4 mL)	Cl_2CH_2	MeOH:NH_4OH (95:5)	MeOH
Total recovery (CSSP+GSSP) (%)	14.9	95.8	38.3

Table S2. Statistical and performance characteristics for the SPE-UHPLC-MS/MS proposed method for the determination of PTSO residues.

Matrix	Linear dynamic range (μg kg^{-1})	R^2	LOD (μg kg^{-1})	LOQ (μg kg^{-1})
Battery cage farming eggs	211.3–25000	0.994	63.4	211.3

Table S3. Precision (% RSD of peak areas) of the proposed method for the monitoring of PTSO residues in eggs.

Parameter	Repeatability (n = 9)	Intermediate precision (n = 15)
Level	Battery cage farming	Battery cage farming
0.25 mg kg^{-1}	3.9	5.9
2.5 mg kg^{-1}	5.2	7.1
25 mg kg^{-1}	4.1	7.7

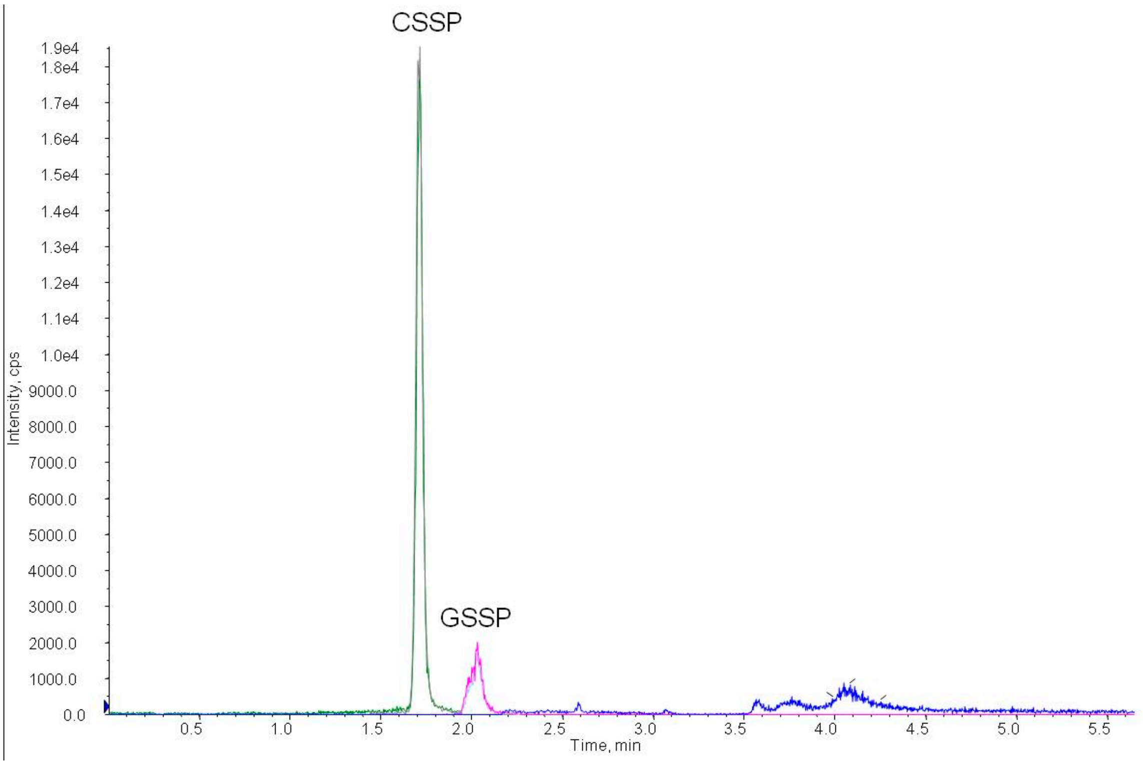

Figure S1. Chromatogram of an egg sample from a free farming hen spiked with 2.5 mg kg^{-1} of PTSO and subjected to the proposed method.
